# The Impact of COVID-19 on Multidisciplinary Care Delivery to Children with Cerebral Palsy and Other Neuromuscular Complex Chronic Conditions

**DOI:** 10.3390/children10091555

**Published:** 2023-09-15

**Authors:** Hillary Brenda Nguyen, Neha Mulpuri, Danielle Cook, Michael Greenberg, M. Wade Shrader, Ryan Sanborn, Kishore Mulpuri, Benjamin J. Shore

**Affiliations:** 1 Harvard Medical School, Boston, MA 02115, USA; hillary.nguyen@childrens.harvard.edu; 2 Boston Children’s Hospital, Department of Orthopedic Surgery, Boston, MA 02115, USA; danielle.cook@childrens.harvard.edu (D.C.); ryan.sanborn@childrens.harvard.edu (R.S.); 3 Department of Internal Medicine, University of Texas Southwestern Medical Center, Dallas, TX 75390, USA; 4 Department of Orthopedic Surgery, Alfred I. Dupont Institute, Wilmington, DE 19803, USA; 5 Department of Orthopaedics, BC Children’s Hospital, University of British Columbia, Vancouver, BC V6H3NA, Canada

**Keywords:** COVID-19, cerebral palsy, neuromuscular conditions, pediatrics, orthopedics

## Abstract

The COVID-19 pandemic has caused unprecedented challenges in the care of children with cerebral palsy (CP) and other neuromuscular complex chronic conditions (NCCCs). The purpose of this study is to explore the direct impact of the COVID-19 pandemic on healthcare delivery. From May to August 2020, medical professionals caring for CP and NCCC patients across multiple countries and disciplines completed a self-administered cross-sectional survey comparing practices before and during the COVID-19 pandemic. Of the 79 healthcare workers from eight countries who participated—predominantly pediatric orthopedic surgeons (32%), pediatricians (30%), and pediatric physiatrists (23%)—most of them felt that caring for NCCC patients during the pandemic presented unique difficulties, and they reported a significant decrease in the in-person NCCC clinic volume (*p* < 0.001), multidisciplinary appointments (*p* < 0.001), surgical cases (*p* = 0.008), and botulinum toxin/phenol injections. Most providers affirmed that institutional guidelines for perioperative emergent/urgent and elective procedures, workplace settings, and technology were modified to accommodate the ongoing public health crisis. The usage of telemedicine significantly increased for NCCC patient visits (*p* < 0.001). During the COVID-19 pandemic, many children with NCCCs lost access to routine, multidisciplinary care. Telemedicine became an integral part of communication and management. In the setting of the COVID-19 pandemic and with the threat of future healthcare disruptions, these data lay the foundation for trending the evolution of healthcare delivery and accelerating best practice guidelines for children with CP and NCCCs.

## 1. Introduction

The coronavirus (COVID-19) pandemic has posed an unprecedented burden to health delivery systems globally. Since December 2019, COVID-19 has infected millions worldwide, dramatically forcing billions of people to practice social distancing [1]. At an institutional level, the pandemic has disrupted outpatient care and elective surgeries, driving healthcare providers to quickly adapt to new guidelines for delivering care. A recent international survey in early 2020 and 2021 found that most general pediatric orthopedic surgeons paused all elective procedures, reported a decrease in the average number of weekly surgeries and elective outpatient appointments, and increased the usage of virtual modes of communication for the first time [2].

Given that individualized, longitudinal, and intensive treatment is crucial to children with chronic complex conditions (CCCs), COVID-19 has presented a tremendous, ongoing hurdle to their care. A CCC is defined as any medical condition that is expected to last at least 12 months, involves at least one organ system requiring subspecialty care, and warrants hospitalization in a tertiary care center [3]. Neuromuscular chronic complex conditions (NCCCs)—one of 12 CCC categories—encompass cerebral palsy (CP), spina bifida, brain malformations, muscular dystrophy, and seizure disorders [4]. Many patients with CP rely on frequent medical appointments and therapies to combat progressive muscle spasticity and contracture, in addition to orthopedic surveillance and surgical intervention to prevent the progression of neuromuscular hip dysplasia.

While sparse, the extant literature suggests that the COVID-19 pandemic has taken a toll on children with CP and their caregivers. Similarly, clinicians caring for children with CP have experienced significant impacts on their ability to provide timely and effective care. Overall, children with CP were found to have worse mobility, physical function, muscle cramps, pain, spasticity, and quality of life scores due to delayed botulinum toxin administration, reduced therapies, and a lack of access to support [5,6,7,8]. Caregivers reported decreased routine follow-up and physical rehabilitation sessions, with a significant decline in caregivers’ physical and mental quality of life [6,7,9,10]. Telerehabilitation and online–offline hybrid exercise programs have proven to be somewhat effective as temporizing measures, though they are limited in improving gross motor function, well-being, and reintegration to normal living in children with CP [11,12,13].

Therefore, the purpose of this study is to investigate COVID-19-related challenges and modifications in healthcare practice, delivery, and resource utilization for children with NCCCs. To our knowledge, this is the first international study surveying multidisciplinary pediatric practitioners of children with NCCCs to better understand the varying degrees to which the pandemic has changed care within each specialty, and by extension, impacted the health of children with NCCCs.

## 2. Materials and Methods

### 2.1. Survey Development

Adapted from a prior questionnaire created by Gibbard et al. to assess the impact of the COVID-19 pandemic on the practice of general pediatric orthopedic surgeons [2], our current survey comprised open- and closed-ended questions on provider demographics, clinical background, pediatric neuromuscular care before and during the pandemic, and clinic/hospital guidelines for procedures and orthopedic surgeries.

### 2.2. Survey Distribution

After Institutional Review Board (IRB) approval, an electronic REDCap version of the survey was administered between May and August 2020 to an international group of pediatric medical professionals who cared for children with NCCCs [14,15]. The survey was distributed using national LISTSERV, Twitter, and national society chat rooms among members of the American Academy of Cerebral Palsy and Developmental Medicine (AACPDM) and other international academic medical networks utilized by the Cerebral Palsy Center at our institution. Repeat emails were sent to constituents to encourage participation. Inclusion criteria included English-speaking medical professionals (MD, NP, RN, PA, PT, OT, etc.) associated with AACPDM who treat patients with CP and were currently practicing during the COVID-19 pandemic.

### 2.3. Statistical Analysis

Provider demographic and clinical data were described in tabular form. Categorical variables were summarized as frequencies and percentages. McNemar tests were utilized to determine if there were any differences in visit options before COVID-19 pandemic and during COVID-19 pandemic. *p*-values less than 0.05 were considered significant. Themes from the qualitative responses were reviewed and summarized.

## 3. Results

### 3.1. Demographics

A total of 79 responses were received between May and August 2020 from providers across eight countries (Table 1). The respondents were primarily pediatric orthopedic surgeons (25/79, 32%), pediatricians (24/79, 30%), and pediatric physiatrists (18/79, 23%), and 66% had over 10 years of experience since graduating from residency/professional training (50/76). Most respondents were 100% pediatrics-based (62/77, 81%) with a significant focus on neuromuscular disease (54/77, 70%). Over half of all providers, regardless of medical profession, completed more advanced “fellowship” training, covering care for children with NCCC, such as pediatric orthopedics, pediatric rehabilitation medicine, neurodevelopmental disabilities, and pediatric complex care (45/77, 58%).

### 3.2. Clinical Practice

Most respondents (47/79, 59%) felt that it was especially difficult to provide effective care to children with NCCCs during the pandemic, though a minority (31/79, 39%) reported that it was equally as difficult to treat both NCCC and non-NCCC patients alike. Fewer respondents (21/78, 27%) felt that the pandemic affected their ability to provide urgent and emergent care for children with NCCCs. Given that all elective procedures were almost universally stopped by the time the survey was conducted, most providers decided to take a break and resume treatment once the pandemic eased and elective cases were permitted again (51/72, 71%), while 24% opted to book these cases as “semi-urgent” and requested special permission from hospital administration on a case-by-case basis (17/72).

**Figure 1:**
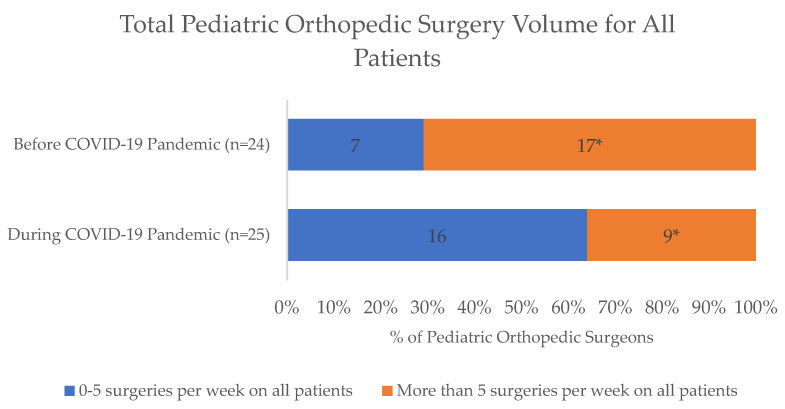
Comparison of the total number of surgeries performed on all patients during the COVID-19 pandemic by pediatric orthopedic surgeons. * Significantly fewer pediatric orthopedic surgeons were performing more than 5 total surgeries per week on all patients during the pandemic compared to before the pandemic (9/25 vs. 17/24; *p* = 0.003).

**Figure 2:**
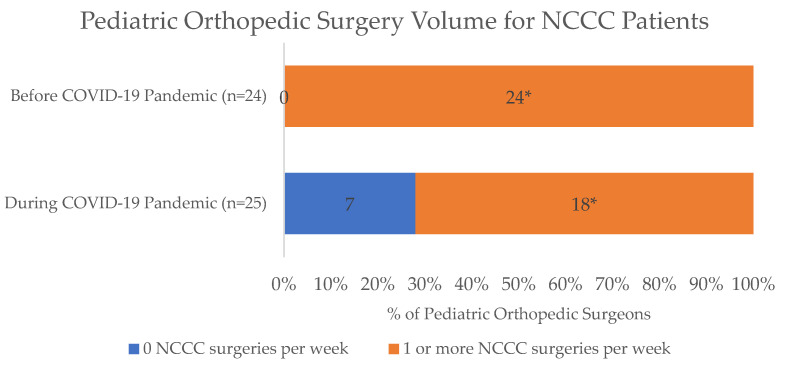
Comparison of the number of surgeries performed specifically on NCCC patients during the COVID-19 pandemic by pediatric orthopedic surgeons. * Significantly fewer pediatric orthopedic surgeons were operating weekly on NCCC patients during the pandemic compared to before the pandemic (18/25 vs. 24/24; *p* = 0.008).

For surgical care, most respondents were aware that elective surgeries at their respective institutions had been deferred since March 2020 (73/77, 95%). Among pediatric orthopedic surgeons (*n* = 25), 68% (17/25) were performing both elective and urgent/emergent procedures, 28% (7/25) were performing only urgent/emergent procedures, and only 4% (1/25) were exclusively performing elective procedures during the pandemic compared to before the pandemic, when most respondents (20/24, 83%) were performing exclusively elective procedures and 17% (4/24) performed urgent/emergent procedures. A significantly lower percentage of orthopedic surgeons reported performing greater than five surgeries per week on all patients at the peak of the pandemic in April 2020 (36%, 9/25) relative to prior to the pandemic (17/24, 71%; *p* = 0.003). Furthermore, while all (100%, 24/24) orthopedic surgeons performed at least one surgery on NCCC patients per week prior to the pandemic, only 72% (18/25) continued at this rate during the pandemic (*p* = 0.008), with seven orthopedic surgeons completely stopping surgeries for NCCC patients altogether (Figure 1 and Figure 2).

**Figure 3:**
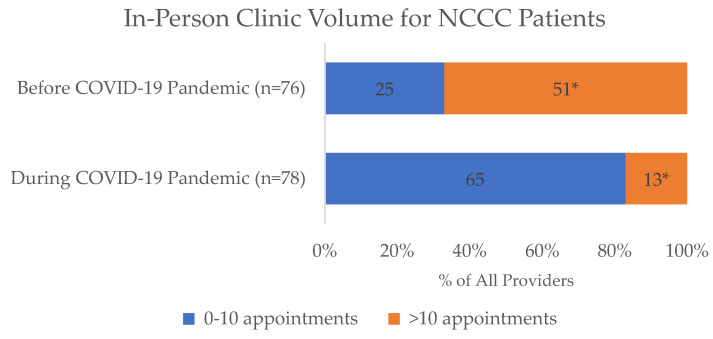
Change in the number of in-person clinic appointments for NCCC patients during the COVID-19 pandemic. * While most providers (51/76, 67%) had more than 10 NCCC appointments prior to the pandemic, significantly fewer (13/78, 17%) were able to retain that volume during the pandemic (*p* < 0.001).

For outpatient care, many providers reported that routine clinic appointments also stopped across all hospital and clinic settings (64/78, 82%) by mid-April 2020. At the height of the pandemic, most providers (44/79, 56%) did not continue to see NCCC patients in person. There was a sharp decrease in providers who had more than 10 in-person NCCC clinic appointments per week from before the pandemic (51/76, 67%) to during the pandemic (13/78, 17%; *p* < 0.001) (Figure 3). Additionally, only 18% providers (14/77) continued in-person multidisciplinary appointments during the pandemic compared to 82% (62/76) prior to the COVID-19 pandemic (*p* < 0.001). About half of the providers (38/79, 48%) reported that their patients were no longer receiving in-person physical or occupational therapy, though most (70/78, 90%) said that their patients were still receiving some therapy (e.g., speech) via telehealth.

In March 2020 of the pandemic, 63% (22/35) and 84% (27/32) of providers stopped administering botulinum toxin/phenol injections in the clinic and operating room, respectively, with an expected date of resumption of 2–4 months later. Reassuringly, braces continued to be modified (56/76, 74%) generally in the following settings: hospital/clinic-based orthotic clinic, private orthotics office, orthotic house call, or other.

### 3.3. Employer and Professional Society Guidelines

At the time of the survey, almost all providers (71/77, 92%) said that their workplace had an Emergency Operations Center/COVID-19 Task Force disseminating daily COVID-19 updates (61/76, 80%) and received guidance from their national professional society (64/76, 84%). A majority of respondents (65/73, 89%) indicated that their respective hospitals provided guidelines regarding emergency and elective surgeries on COVID-19-positive patients. Nearly all providers (71/74, 96%) reported that hospitals were routinely screening patients prior to surgery, personal protective equipment (PPE) was provided by their institutions (68/74, 92%), clinicians were not expected to procure PPE at their own cost (73/77, 95%), and they were not recruited to assist in non-subspecialty trained care in the intensive care unit or the emergency department (ED) (69/79, 87%).

While nearly everyone (70/77, 91%) had to rearrange their work practice (e.g., limited physical contact and no X-rays), most providers (47/77, 61%) were not required to go to the hospital every day during the pandemic. About half (40/76, 53%) of the hospitals/clinics had back-up personnel to perform duties in case the providers were sick, with teams to provide on-call services (39/65, 60%).

### 3.4. Technology

Prior to the pandemic, most providers had never used telemedicine for patient appointments (51/75, 68%) due to a lack of hospital/clinic approval (26/49, 53%), unavailability (8/49, 16%), provider preference (7/49, 14%), patient preference (1/49, 2%), or other reasons (7/49, 14%). During the pandemic, almost all providers (75/78, 96%) adopted virtual clinical platforms, which was significantly higher than prior to the pandemic (24/75, 32%; *p* < 0.001). Most providers reported that their medical council/state licensure relaxed the Health Insurance Portability and Accountability Act (HIPAA) compliance for the duration of the pandemic (43/79, 54%), and many (43/74, 58%) saw virtual patients in additional jurisdictions as a result. Almost all providers stated that virtual visits were permitted by their medical council/state legislature (74/78, 95%).

Popular types of NCCC telemedicine appointments during the pandemic were follow-ups (72/75, 96%) and new patient visits (41/75, 55%). Post-operative visits (28/75, 37%), gait lab/test result reviews (19/75, 25%), and other visit types (6/75, 8%) were less common. Most providers were able to hold multidisciplinary appointments virtually (46/75, 61%). Of those utilizing telemedicine during the pandemic, 74% had 10 or fewer virtual visits per week (54/73), while just 26% of providers (19/73) had greater than 10 visits weekly. Although more than half of the providers (40/73, 55%) were not able to provide scanned written prescriptions during virtual visits, nearly everyone (67/74, 91%) expressed being able to bill. Of those who said they were able to bill, just over half were able to charge the same amount as in-person visits (34/67, 51%), while 28% were billed for less (19/67, 28%), and others did not know (14/67, 21%).

Challenges were frequently encountered when assessing NCCC patients via telehealth (51/73, 70%), especially with physical exams, which were acceptable for general assessments (incisions and overall appearance) but more limited for spasticity, tone, strength, range of motion, gait, orthotic fit, anthropometrics, and subtle findings in conditions such as scoliosis and hip dysplasia. The participants’ qualitative responses revealed that providers had to draw from patient, family, and caregiver participation to assist with the physical exam, which was less reliable and feasible. Some providers even sent patients to the ED for in-person exams regarding issues that were otherwise appropriate for outpatient visits. 

Many providers also highlighted communication struggles with patients and families over virtual visits. Relying on families to report patient progress can be less accurate than in-person observation, and verbally explaining how to perform certain exam maneuvers virtually can be unwieldy. An even greater burden was placed on families requiring interpreters.

Another theme among the qualitative data was the challenge of finding a proper setting for telehealth/virtual visits. It was arduous for families and patients to access quiet, private locations without distractions. For patients necessitating imaging, providers had to either forgo or separately coordinate radiology visits if possible. A final common obstacle was the technology itself, which was often slow and cumbersome, increasing fatigue for both parents and providers. Learning the skills to be technologically savvy and acquiring adequate electronic resources/equipment (e.g., a laptop or cellphone), internet, and video/audio quality were barriers. For providers, virtual visits were sometimes reported to be less efficient and demanded more time/energy spent on navigating technology, as well as reviewing and documenting supplemental media.

### 3.5. Provider Connection and Wellness

Most providers (73/78, 94%) continued formal meetings with colleagues during the pandemic using mostly video calls—sometimes phone calls—on Zoom, Microsoft Teams, Skype, and other platforms (e.g., Webex, BlueJeans, and Attend Everywhere). Staff wellness activities were implemented at most hospitals, including chaplain “check-ins”, peer-led counseling, free food, mindfulness, and compassion rounds (48/76, 63%). The majority felt that their administration cared about their well-being (68/76, 89%) and felt safe at work (67/77, 87%).

## 4. Discussion

The multidisciplinary, collaborative nature of caring for children with NCCCs makes the restrictions related to the COVID-19 pandemic particularly problematic. At the beginning of the pandemic, significant changes to healthcare delivery led to negative outcomes for children with NCCCs and their caregivers, as reported in prior studies [5,6,7,8,9,10]. The findings from our survey are consistent with other published studies in the literature, which have described that children with physical disabilities experienced interruptions in their medical follow-up and rehabilitation [16]. Consequently, the well-being and mental and social health of these children were negatively impacted as well [17]. Given that children with physical disabilities are at a higher risk for developing mental health symptoms [18] and sedentary behavior [19], a lack of social interaction and opportunities for physical activity can be detrimental to their psychological well-being and functional ability. Now, documenting the perspective of their providers, our study results support these findings, with most providers acknowledging that it was more difficult to care for this patient population during the pandemic, though nearly 40% of clinicians reported similar difficulties in treating all children regardless of NCCC status.

Institutional and regional regulations, such as restrictions on elective procedures and in-person clinic visits, in addition to concerns about contracting COVID-19, were common challenges. Given these newfound obstacles, nearly all providers adopted a telemedicine platform. Despite its shortcomings, telemedicine played a large role in ensuring the continuity of care for NCCC patients outside of the standard settings. Though unable to completely substitute face-to-face clinic visits, telemedicine can be strategically used as an adjunct to in-person clinic visits. If the focus on telemedicine continues, we recommend an additional development and validation of components of the physical exam, which can be completed on a telemedicine platform (e.g., gait, range of motion, scoliosis, etc.). Informational videos and documents are needed for providers and parents/patients to improve the telemedicine visit utility. Patients and families should also be asked about their proficiency in and access to technological equipment, and should be referred for adequate assistance if deemed to be disadvantaged or lower-resourced. As the care for children with NCCCs requires a unique and more nuanced approach, this needs assessment should be made a priority to optimize the value of each telemedicine appointment.

The strengths of this study are twofold: it informs future best practice guidelines in the setting of future pandemics and highlights areas for continued investigation/improvement. Given that scientists estimate that there are approximately 1.7 million undiscovered viruses, there is a risk for another deadly, easily transmissible pathogen [20]. The new monkeypox outbreak of 2022–2023 is one such example [21]. To avoid repeating similar disruptions in healthcare delivery, steps can be taken now to mold resilient systems that facilitate the continuity of care for children with NCCCs. Applying the science of quality improvement (e.g., plan–do–study–act cycles) to the pressurized circumstances of the COVID-19 pandemic can ensure a methodical, sensitive, and pragmatic approach for tackling procedural and structural changes [22,23]. For patients sustaining unexpected setbacks during COVID-19, the medical community has a duty to ensure that these children are given opportunities to thrive in this new norm. This includes adopting a multi-pronged, coordinated approach that tackles a balance between the risk of the spread of infectious diseases and the overall health goals of the patient, alongside dedicated support for their family and caregivers. At the very least, maintaining contact through virtual visits and remote rehabilitation can ensure that patients are not lost to follow-up. These lessons can also be applied to children with other CCCs, as well as to lower-resource settings that are vulnerable to these disruptions.

This study is not without limitations. Recall and selection biases are inherent in self-reported responses. Additionally, though this survey intended to gather international experiences, having a smaller sample size and few responses outside of the United States limits its generalizability. Furthermore, many advances in COVID-19 response/treatment have been made since the study data were collected in 2020, warranting a more updated look; however, capturing the same respondents at a second time point was not feasible for this study. Finally, while our study demonstrates that most providers had access to staff wellness programs, it is unclear how these have influenced morale, mental health, and performance. Provider burnout, which was amplified during the pandemic, impacts both occupational workers and patients, with recent reviews suggesting a link between physician burnout and negative outcomes for patient care, such as increased medical errors [24]. Additional exploration to promote a healthier work environment will ultimately improve the quality of care delivered to children with NCCCs during these stressors.

## 5. Conclusions

Our study reveals that practices across multiple disciplines may have suffered during the peak of the COVID-19 pandemic, with providers perceiving that suboptimal care for children with CP and NCCCs transpired. As we transition to living with the reality that similar pandemics are possible, rigorously examining and understanding the barriers from the COVID-19 pandemic can inform the development of sustainable solutions to prevent our healthcare system from coming to a halt again and improving the overall care of children with NCCCs.

## Figures and Tables

**Table 1:** Summary of the demographics, type of practice, and setting of practice for the study participants.

Demographics	N	Freq.	(%)
Medical profession	79		
Orthopedic Surgeon		25	(32%)
Physiatrist		18	(23%)
Subspecialty Pediatrician		24	(30%)
Nurse Practitioner		5	(6%)
Occupational Therapist		1	(1%)
Physical Therapist		1	(1%)
Other		5	(6%)
Pulmonologist		1	
Dietitian		1	
Registered Nurse		1	
Unspecified		2	
Number of years post residency/professional training	76		
>20 years		29	(38%)
16–20 years		11	(14%)
11–15 years		10	(13%)
6–10 years		19	(25%)
0–5 years		7	(9%)
Fellowship training that included the care of children with neuromuscular disease	77		
Yes		45	(58%)
No		26	(34%)
N/A		6	(8%)
**Type of Practice**	**N**	**Freq.**	**(%)**
Percentage of practice that is pediatric	77		
100%		62	(81%)
75%		12	(16%)
50%		1	(1%)
25%		2	(3%)
Percentage of practice that involves pediatric neuromuscular disorders	77		
100%		10	(13%)
75%		26	(34%)
50%		18	(23%)
25%		22	(29%)
0%		1	(1%)
Country of practice	75		
United States of America		64	(85%)
Canada		4	(5%)
Australia		2	(3%)
United Kingdom		1	(1%)
India		1	(1%)
Switzerland		1	(1%)
Qatar		1	(1%)
Brazil		1	(1%)
Practice setting	77		
Metro (Population > 200,000)		61	(79%)
Urban (Population 40,001–199,999)		11	(14%)
Rural (Population 10,001–40,000)		3	(4%)
Remote (Population 1–10,000)		2	(3%)
Practice location	77		
General hospital managing adult and pediatric patients		3	(4%)
Combined private and hospital practice		4	(5%)
Tertiary care center managing both adult and pediatric patients		17	(22%)
Pediatric specialty hospital		47	(61%)
Private clinic/nursing home or independent clinic		2	(3%)
Non-hospital, community service		3	(4%)
Other (military, freelance, orthopedic urgent care)		1	(1%)

## Data Availability

The data presented in this study are available upon request from the corresponding authors. The data are not publicly available due to privacy reasons.
